# CrossSwap: A shape-aware identity cross-attention face swapping framework

**DOI:** 10.1371/journal.pone.0349356

**Published:** 2026-05-14

**Authors:** Hojun Seo, Junyeoung Ahn, Byungseok Kang

**Affiliations:** 1 dob studio Inc., Donggyo-ro, Mapo-gu, Seoul, Republic of Korea; 2 GenON Inc., Nambusunhwan-ro, Gangnam-gu, Seoul, Republic of Korea; FEI: Centro Universitario da FEI, BRAZIL

## Abstract

Face swap technology is a field of great interest within generative AI. Users can create a new image by swapping the face in the image they take with another face of their choice. However, existing face swap technologies often produce unnatural facial images, losing the identity of the original source image. In this paper, we propose the CrossSwap model to solve these shortcomings. CrossSwap includes scale-adaptive cross-attention blocks that enable smoother swapping between the source and the target face. Leveraging Shape-Aware Identity Extractor with such architecture, CrossSwap preserves the facial shape of the source face and rich identity features while maintaining properties such as the pose of the target face. We evaluate our method using various metrics including Identity Retention (ID), Fréchet Inception Distance (FID), Pose Errors (Pose) and Expressions Errors (EXP) with three recently introduced methodologies. The quantitative experimental results showed that 3.8 Pose(1^st^), 0.276 ID(2^nd^), 11.5 FID(2^nd^), and 1.37 EXP(1^st^), respectively. The proposed method achieved the highest scores for Pose and EXP.

## 1. Introduction

Face swapping is a technology that replaces a face in an original image or video with a new face chosen by the user. Recent face swap results have implemented subtle changes in facial expressions and even subtle movements of facial muscles. It can also automatically reconstruct the background and even project realistic shadows, fooling people’s eyes. Face swapping, which is the task of swapping faces between source and target faces, must be carefully imported into the target face while preserving the identity information of the source.

Various types of face swap technologies have appeared so far. The initial face swap model trained an auto-encoder for each source and target face and then swapped the decoder so that the output face included both the source and target faces [1,2]. However, this architecture has a disadvantage in that it needs to be retrained whenever the source and target people change because it varies depending on the person. To solve this problem, face swapping architectures that use identity extractors have emerged [3,4]. However, these architectures also failed to retain most of the identity information of the original. This is because existing extractor models were not fundamentally trained to accurately represent the identity information of the input image.

Another problem with the existing face swap models is that the identity information of the source face often overwhelms the target information due to the conflicting design of the loss function to simultaneously minimize the identity loss and the attribute loss. This means that even a well-extracted identity embedding of the source face may be useless if the information is lost. Since then, many studies have been conducted to maintain the identity of the original face, but the results have not been very good. What they did not consider is the preservation of the outline of the source face, which is an important factor for the fine face swap results.

In this context, we rethink the role of identity embedding as a well-blended helper rather than the source identity embedding through a novel design of a cross-attention-based architecture. It is responsible for fast convergence during training and smoothly blends the facial shape of the source image and the richer identity information of the source image with the attributes of the target image. The most important module of our proposed model is the cross-attention generator. This module effectively learns and infers data to maintain the original identity in the output.

The purpose of this study is to generate more natural faces using face swap technology. There are qualitative aspects that are natural to the human eye and quantitative aspects that are measured by ID, FID, Pose error, and expressions errors. We compare its performance with four recently published face swap models through quantitative and qualitative experiments. The experimental results show that our model outperforms the existing models in most areas. Furthermore, we experiment to do ablation study to understand the contribution of each component of proposed model. The ‘Attention’ mechanism is an important concept in deep learning models, especially in the transformer model in the field of Natural Language Processing (NLP). Cross-attention is an attention mechanism used to learn and utilize the relationship between two different input sequences. This is the first study to apply the Cross-attention technique to the Face Swap method. The experimental results verified that this method is very effective for face swap. The cross-attention method we proposed has the following limitations. As the sequence length increases, the attention weights and computational complexity increase exponentially, resulting in a significant increase in computational cost and large memory requirements when processing very long sequences. The attention mechanism itself requires a large amount of memory to load and process all data in a long sequence, which can lead to an out-of-memory (OOM) problem.

## 2. Related works

### 2.1. Subsection identity embedding

Face swapping, a task of swapping faces between source face and target face, needs to meticulously bring source’s identity information (e.g., eyes, mouth, etc.) into target face with its attributes preserved (e.g., hairstyle, ears, etc.). There are a few trials that train auto-encoders for each source and target faces then swap their decoders [1,2] such that output faces contain both source and target faces. But such architectures are person-dependent that need to be re-trained whenever source and target people change. In addition, absence of explicit separation between attribute feature and identity feature leads to entanglement in latent space and difficulty in convergence of training. Thus, face swapping architectures using identity extractors emerge and most of them [3,4] choose to use ArcFace [5] and CosFace [6] as an identity extractor. FlowFace [7] and FlowFace++ [8] adopt Masked AutoEncoder (MAE) [9] as an identity extractor, pointing out [5,6] are originally designed for face recognition and therefore do not contain identity information of face properly. ExtSwap [10] and StyleSwap [11] use Style-Based Generative Adversarial Network (StyleGAN) [12] generator, regarding identity swapping as style transferring by using identity embedding as style codes. In such approaches, various identity embedding [13,14] are adopted but their success is due to StyleGAN achieving high-fidelity generative power [15]. Thus, none of these methods deals with improving identity embedding itself, only trying to replace them with new ones. Still, the problem is that such extractor models are not fundamentally trained to represent identity information of input image [14].

Another issue is that, due to the conflicting design of loss functions to simultaneously minimize identity loss and attribute loss, identity information of source face used to eventually be overwhelmed by target information. This means even well-extracted identity embedding of source can become of no use losing its information.

Based on these contexts, we re-think of the role of identity embedding as a well-blending helper rather than source identity embedding with a new design of cross-attention-based architecture. It oversees fast convergence during training and preserving facial shape of source image and richer identity information of source image with attributes of target image smoothly mixed. DiffSwap [16] points out such implicit injection of the shape information cannot produce satisfactory results when the source face shape and the target face shape differ by a large margin. Our cross-attention-based architecture mitigates this issue by meticulously attending to both the source and the target feature maps sufficiently and adaptively lessening such discrepancy. SmoothSwap [17] takes smoothness of identity embedding into consideration via their novel architecture for retaining smooth identity embedding. Their contribution, however, is not about facial outlines of source faces that are also important ingredients in fine-grained face swapping results.

### 2.2. Attribute feature

Combining a source face and a target face has been evolved mainly into two ways; Source-based methods and target-based methods. Source-based methods modify attribute features of target face into source face then blend the source face to target background image. An early method [18] employs 3D Morphable model (3DMM) [19] to capture expression and posture then blend them. However, the method is often limited in handling lighting and skin color. [20] extracts 2D facial landmarks of source and target faces then computes 3D pose and modifies 3D shape by using the landmarks, which is then segmented. Finally, the source face is warped onto the target face as it is, which is finalized with blending. Limitation of the method is the unnatural lighting of the stitched face of the warped source face and the target face due to the off-the-shelf method employed without optimization. Face Swapping GAN (FSGAN) [21] devises a two-stage architecture where target face pose and expression are transferred to a source face and then the source face blends into the target background image.

FSGAN has made significant progress in the field of face swapping and face reenactment. It has clear advantages and limitations as well as powerful functions. Many existing deepfake models had to individually train models with data of specific people to swap faces between two specific people. FSGAN can swap or reenact faces of new people that have not been seen during the training process in near real time without these restrictions. This is because the model learned by generalizing the universal structure, expression, and pose deformation of the face rather than the unique features of a specific person. This maximizes the convenience of practical use. No matter how advanced it is, deep-fake technology is not perfect. FSGAN can also have unnatural blending boundaries around the facial contour, or cases where hair, glasses, and accessories are not properly handled. Artifacts can be more noticeable in videos with complex lighting conditions, extreme poses, and fast movements.

These Target-based methods typically employ neural networks to learn attribute features and to inject source identity embedding into the attribute features. FaceShifter [3] adopts off-the-shelf model ArcFace [5] to extract source identity embedding and multi-level attributes encoder to learn robust multi-spatial attributes of target face. Then source identity embedding is fused into the target attributes. The method generates swapped faces with high fidelity with help of their own post-processing network. SimSwap [15] seeks to separate identity information from the decoder. It first extracts target face feature which is fed to identity injection module to change identity information of target face towards of source face. The main goal of SimSwap is to seamlessly transplant the identity of a source face onto a target face while preserving the original facial features (expression, pose, gaze direction, etc.) as much as possible. The model is also applicable to video, and attempts to maintain consistency between frames, resulting in a flicker-free result.

Profound research has been conducted on utilizing StyleGAN [22] in face swapping tasks due to the StyleGANs’ ability to manipulate given features in granularity by tweaking latent codes. [11,23–30] utilize pre-trained StyleGAN to represent the source identity with latent representations with their novel methods. These representations are injected to the target face to intrinsically mix target attribute and source identity. StyleGAN methods showcase superb results in face swapping. The biggest advantage of StyleGAN is the amazing quality and realism of the images generated. It generates very natural images that are difficult for the human eye to distinguish from real images. This is especially noticeable in the generation of face images, and it creates faces of “people who do not exist in this world” very convincingly. However, it is not completely free from one of the inherent problems of GAN models, learning instability. In complex datasets or certain settings, problems such as mode collapse may occur, which may limit the diversity of generated images.

### 2.3. Cross attention mechanism

Attention mechanism has been proven to provide excellent performance to learn representations for objective functions in various domains. In the case of a face swapping task, learning where to learn to swap between a face of target image and a face of source image is the crucial purpose to which cross attention can be well suited since attention mechanism is originally devised to solve machine translation task where one language is converted into one another. In this sense, translating features of source and target faces is a quite similar task as the original one, which drives us to experiment.

There have been a few attempts to challenge face swapping task with attention mechanism as it is evidently proven to be superb in various neural network fields. FaceTransformer [31] uses cross-attention between source and target faces in three different scales for better representation. It uses separate feature extractors for values than queries and keys. FlowFace++ [8] fuses source and target embedding from MAE [9] by cross-attention mechanism assuming related pixel patches would attend more related identity information. They do not explicitly inject source identity embedding as they assume embedding of the source face that is processed by the masked auto-encoder can capture the equivalent source features. Despite the novel approach devised, it suffers from drastic changes in source face image especially in lighting and posture.

## 3. Proposed methods

**Fig 1:**
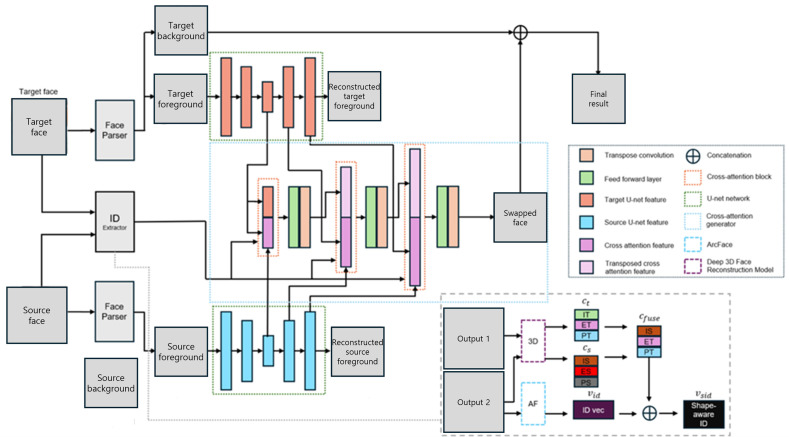
CrossSwap model architecture overview. In details of ID extractor, IT, ET, and PT denote identity, expression, and pose coefficients of target face (IS, ES, and PS for source face).

In general, face swapping task pursues generating a target face naturally fused with source identity. Our paper proposes a novel face swapping method, namely CrossSwap, which generates a swapped face image with consistent source identity using cross attention mechanism along with a shape-aware identity extractor. As illustrated in Fig 1, CrossSwap is a method which is composed of components including source identity extractor, U-net attribute feature extractors for both source and target faces, and cross-attention generator which fuses features from source and target U-net feature extractors and source identity embedding from the source identity extractor. The detailed information will be explained in the coming subsections.

### 3.1. Shape-aware identity extractor

Most face swapping methods often lead to unsatisfactory swapped images (e.g., inconsistent facial outlines from the source face) due to insufficient source information in identity embedding and coarse blending between source identity and target attributes. To tackle such issues, we adopt a shape-aware identity extractor for better identity preservation, inspired by HifiFace [32]. ArcFace [5] has been widely used as an identity embedding extractor, but we find it alone is not sufficient to represent source identity information since it is originally trained for face recognition task [8,14]. Moreover, [5] lacks the power to perfectly separate multiple properties where some belong to source face and the other to target, which gets severer due to colliding design of identity loss for source face and attribute loss for target face. Shape-aware identity embedding mitigates such drawbacks of ArcFace [5] embedding by additionally concatenating facial information obtained by 3D face reconstruction model [33].

Such concatenation has two major advantages. Firstly, due to additional information from the different model, it is provided with generalizability power. Secondly, richer information that leads to faster convergence of training steps and improvement in performance. Specifically, we regress 3D coefficients of each source and target face with the 3D reconstruction model [33]. What we want from source’s 3D coefficients among identity, expression, and pose is only the identity coefficients while we’d like to keep of expression and pose of target face. Thus, we mix those coefficients such that they compose a semantically swapped 3D face but still in vector embedding format. To leverage such information with the identity embedding of ArcFace, we concatenate two vectors to produce the final identity embedding which is depicted in Fig 1. The original shape loss introduced in HifiFace [32] rather makes training unstable due to its redundant use of shape information after a couple of experiments. Therefore, we only adopt one among two terms for shape-awareness to preserve source image’s identity and achieve stable training at the same time, namely simplified shape loss. We will elaborate more on this in the 3.5 objective functions section.

### 3.2. Identity injection via AdaIN ResBlock

To successfully fuse source face identity embedding with target face attribute features, we adopt Adaptive Instance Normalization Residual Block, namely AdaIN ResBlock. AdaIN [34] is a variation of Instance Normalization [35] adopted by StyleGAN [22], which normalizes feature maps and de-normalize it with statistics of style code without any learnable parameters for style transferring. In our method, identity embedding is regarded as style vectors such that the identity information is well infused into target attribute features. Since single AdaIN operation may not be sufficient to train and infuse identity information well, residual block that comprises multiple convolution layers with skip connection is introduced for wide-enough receptive field towards better feature learning. We also carried out some experiments to compare AdaIN ResBlock to mere concatenation and figure out AdaIN ResBlock brings significant improvement both in terms of ID metric scores and perceptual image quality. Moreover, we find that the change of the number of identity injections through AdaIN ResBlock also makes significant difference in swapped image quality. Please refer to the Experiments section.

AdaIN originally gets scale and bias from multi-dimensional style vector while our identity embedding is one-dimensional vector. To handle this, we pass it to two different Multilayer Perceptron (MLP) to increase dimensionality, inspired by the fact that key and value are obtained from single source changing dimensionality through MLP layers in attention mechanism. By doing so, we indirectly calculate pseudo meaning and variance from each of them.

With cross-attention features and the Identity embedding injection fused together, powerful ID embedding features are forced in generation process which leads to a superb performance in terms of Identity preservation.

### 3.3. U-net attribute feature extractor

Most existing methods only take target attribute features with source identity embedding, which might waste rich learned information of other source face features that could have been exploited. Therefore, we employ U-net networks to learn rich attribute features not only of target face but also of source face to better map representations of faces. We reconstruct source and target input faces through the U-net network as below:


$$\begin{array}{c} {\hat{X}}_{*recon}=Unet\left(X_{*}\right) \end{array}$$ (1)

**Fig 2:**
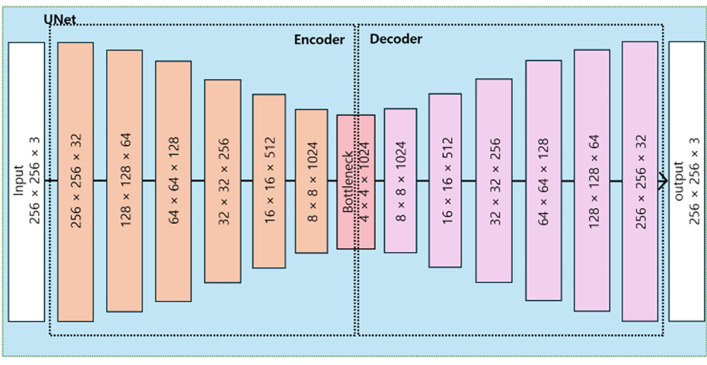
U-net dimensions in CrossSwap.

Where Unet is the U-net network to reconstruct input face, $X_{*}$ is an input image of $X_{\text{t}}$ (target face input) and $X_{\text{s}}$ (source face input), and ${\hat{X}}_{*recon}$ is the reconstructed outcome of $X_{\text{t}}$ or $X_{\text{s}}$ from Unet. Our U-net dimensions in architecture are illustrated below in Fig 2.

### 3.4. Cross-attention generator

**Fig 3:**
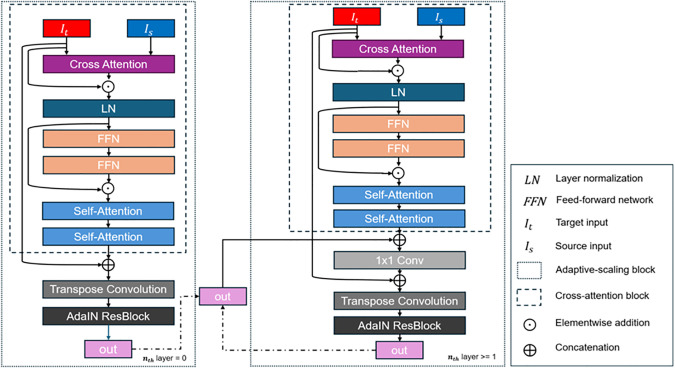
Architecture of cross-attention generator in detail. At layer 0 (the bottleneck feature part), the operations on the left side are used and from layer 1 upwards, right-side operations are implemented.


Learning mappings between source face and target face is an important intrinsic task when it comes to how much we want specific part of target face to attend source face as for identity. To achieve the goal, cross-attention mechanism [36] between source and target faces is employed. We devise cross-attention mechanism as follows. Firstly, we initialize $Q_{*}$, $K_{*}$, and $V_{*}$ vectors. After that, the cross-attention between source and target faces is computed by:


$$\begin{array}{c} CA\left(Q_{t},K_{s}\right)=Softmax\left(\frac{Q_{t}\overset{T}{\underset{s}{K}}}{d_{k}}\right) \end{array}$$ (2)

where $Q_{t}$ is a representation of query vectors of target face, $\overset{T}{\underset{s}{K}}$ is transposed source face key vectors, $d_{k}$ is dimension of key vector, and $CA\left(Q_{t},K_{s}\right)$ denotes cross-attention operation with $Q_{t}$ and $K_{s}$ inputs. The computed CA output as an input goes through more operations, which forms cross-attention block depicted in Fig 3. Firstly, the CA output in (2) is multiplied by source value vectors $V_{s}$ in elementwise manner and then processed via a skip-connection with target feature input as in (3) which outputs $O_{s,t}$. Note that we decided to use target feature input $I_{t}$ in Fig 3, rather than $V_{t}$, which results in slightly better performance in our experiment, which is slightly different from [8].

$$\begin{array}{c} O_{s,t}\;=\;CA*V_{s}\;⊙I_{t} \end{array}$$ (3)

where ⊙ denotes element-wise addition operation. $O_{s,t}$ further goes through a layer normalization [37]. It then is fed into two Feed-Forward Networks (FFN) for expressiveness and dimension manipulation. The output of the FFN is accompanied with features before the FFN layers by another skip-connection. Consecutively, the output is further processed by two self-attention layers which output features of cross attention block.

The cross-attention block is performed in spatial scales of U-net decoder part. To be specific, to generate a swapped face with richer representations on dynamic spatial information, multi-scale features of the U-net decoder part are used as inputs of cross-attention mechanism. As shown in Fig 1, bottleneck features of the U-net of source and target faces are fed into cross-attention block to learn attention mapping features. At the first spatial information part which is the bottleneck dimension, the target’s U-net bottleneck features $n_{th}\;=\;layer\text{0}$ are concatenated to the output features of cross-attention block $n_{th}\;=\;\text{0}$ then are fed into a transpose convolution and AdaIN ResBlock layers, which forms adaptive-scaling block feature ${{out}_{n}(n}_{th}\;=\;\text{0})$ which have the same shape as features of next layer’s cross attention block. Note that at the bottleneck spatial scale, operations are a little bit different from the rest of spatial scales. In all other spatial scales, we concatenate cross-attention block output with the previous spatial output ${out}_{n-\text{1}}$ on the right side of Fig 3 and feed it into a 1x1 convolution layer. It is then concatenated with the target features $I_{t}$ again and processed by a transpose convolution layer to match the spatial shape of next level feature size of the U-net. Finally, the feature is passed into the AdaIN ResBlock where it is normalized and de-normalized with the pseudo mean and variance of an identity vector, having the similar effect of style transfer from target face to source face, which results in ${out}_{n}$. The aforementioned steps (detailed in Fig 3) are repeated until the final output image resolution. Note that the last features are only applied with the transpose convolution layer without cross attention operation for the sake of reduction in Graphics Processing Unit (GPU) operations.

**Fig 4:**
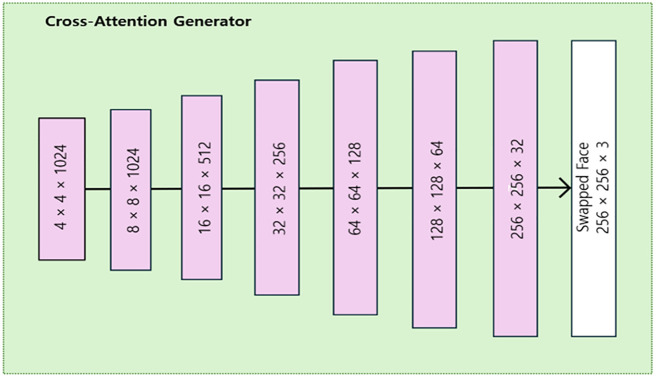
Cross-attention generator dimensions in CrossSwap. Where D denotes the discriminator.

We argue the cross-attention mechanism intensifies source identity blending along with the shape-aware identity extractor which makes our method more robust in terms of identity preservation. The cross-attention generator dimensions are depicted below in Fig 4.

### 3.5. Objective functions

Training CrossSwap follows GANs adversarial training to generate $\hat{Y_{s,t}}$ realistic where $\hat{Y_{s,t}}$ is the outcome of swapped face.

#### 3.5.1. Adversarial loss.

Adversarial loss is implemented with a multi-scale discriminator to capture multi-spatial information [38] and hinge loss to prevent from mode collapsing and to enhance realism.


$$\begin{array}{c} L_{adv}=-E\left[D\left(\hat{Y_{s,t}}\right)\right] \end{array}$$ (4)

#### 3.5.2. Attribute loss.

As we use multi-scale attributes, learning intermediate multi-scale features would enhance the latent representations which improves loss convergence. Therefore, we incorporate attribute loss as follows:


$$\begin{array}{c} L_{attr}=\frac{\text{1}}{l}\sum_{i=\text{1}}^{l}\overset{\text{2}}{\underset{\text{2}}{\left|\left|\overset{attr}{\underset{t[i]}{X}}-\hat{\overset{attr}{\underset{t[i]}{X}}}\right|\right|}} \end{array}$$ (5)

Where $\overset{attr}{\underset{t[i]}{X}}$ is target face attributes at $i_{th}$ intermediate spatial resolution and $\hat{\overset{attr}{\underset{t[i]}{X}}}$ is reconstructed outcome of the target face attributes at $i_{th}$ spatial resolution from the cross-attention generator. Note that the attribute loss does not come from U-net network but cross-attention generator.

#### 3.5.3. Reconstruction loss.

When the source and the target face are from the same face image, the generated face from them should look the same as the target and the source faces. To learn pixel reconstruction capability, we enforce $X_{t}\;=\;X_{s}$ with a small probability. We let $X_{t}\;=\;X_{s}$ in a loose term, which means not only when $X_{t}$ and $X_{s}$ faces are from the same images, but also when $X_{t}$ and $X_{s}$ faces are from the same person but not entirely from the same image if data is applicable [39]. The reconstruction loss is $L_{\text{2}}$ loss as below:


$$\begin{array}{c} L_{rec}=\;\left\{ \begin{array}{cc} \overset{\text{2}}{\underset{\text{2}}{\left\|X_{t}-\;{\hat{Y}}_{s,t}\right\|}} & \;\text{if }X_{s}\in \;person_{i}\text{ and }X_{t}\in \;person_{i}\\ \;\text{0},\; & \;\text{otherwise} \end{array}\right.\; \end{array}$$ (6)

#### 3.5.4. Identity loss.

The identity loss is calculated to induce identity similarity between $X_{s}$ and ${\hat{Y}}_{s,t}$.


$$\begin{array}{c} L_{id}=\text{1}-\text{cos}\left(z_{id}\left(\hat{Y_{s,t}}\right),{\;z}_{id}\left(X_{s}\right)\right) \end{array}$$ (7)

Where $z_{id}$ denotes ArcFace model, cos denotes cosine similarity. $X_{s}$ and ${\hat{Y}}_{s,t}$ mean source face input and generated output from cross-attention generator respectively.

#### 3.5.5. Contrastive loss.

As mentioned earlier, identity extraction is an imperative step for the goal of face swap. To better emphasize identity learning, we employ contrastive face identity loss motivated by [40]. The loss in our method utilizes all the target identity embedding within a mini batch except the case where the source and the target faces are set to the same image as negative sample pairs, and each source embedding is treated as a positive sample pair. Letting $S_{*}=S_{sim\left(\overset{*}{\underset{id}{E}},\;\overset{s,t}{\underset{id}{E}}\right)}/\tau$, the contrastive loss is formulated as:


$$\begin{array}{c} \begin{array}{c} L_{cont}=\;-\text{log}\left(\frac{\text{exp}\left(S_{s}\right)}{\text{exp}\left(S_{t}\right)+\;\sum_{n\;\sim P_{neg}}\text{exp}\left(S_{n}\right)}\right)\; \end{array} \end{array}$$ (8)

Where $\overset{id}{\underset{s}{E}}$, $\overset{id}{\underset{t}{E}}$, and $\overset{id}{\underset{s,t}{E}}$ denote identity embedding of source face, target face and swapped face respectively that are made through ArcFace [5] extractor. $S_{sim}$ represents the normalized dot product operation and $P_{neg}$ denotes the negatives that are sampled from target faces in a mini batch.

#### 3.5.6. Landmark loss.

To ensure consistency of facial alignment in poses and expressions of the target face, we implement regularizing loss for landmarks [41] detected by face parsing model. The loss is formulated between $X_{t}$ and $Y_{s,t}$:


$$\begin{array}{c} L_{lm}=\frac{\text{1}}{n}\overset{\text{2}}{\underset{\text{2}}{\left|\left|F_{lm}\left(X_{t}\right)-F_{lm}\left(\hat{Y_{s,t}}\right)\right|\right|}} \end{array}$$ (9)

Where $F_{lm}$ denotes a face landmark detector.

#### 3.5.7. U-net reconstruction loss.

Representations of intermediate layers in multi-scaled features of U-net networks in our method are crucial to learn cross attention mappings between source and target faces as they come in as inputs of cross attention blocks. To enhance the representations, we devise U-net face reconstruction loss which uses $L_{\text{2}}$ term as:


$$\begin{array}{c} L_{unet}=\overset{\text{2}}{\underset{\text{2}}{\left|\left|X_{input*}-\hat{X_{recon*}}\right|\right|}} \end{array}$$ (10)

#### 3.5.8. Shape loss.

While the main purpose of using identity embedding is to preserve or inject a source image’s identity into the target face, it still needs to have attributes of a target face, which easily conflict with each other. Therefore, it leads to unnatural swapped outputs. To let our shape-aware identity embedding exert its power such that the swapped face becomes smoother and has a shape of a source image, shape loss is designed to implement shape-awareness of identity embedding HifiFace [32]. By regressing $C_{fuse}$ in Fig 1, they generate 3D face model using a mesh renderer, then also generate another 3D face model of swapped image and its intermediate (low resolution) feature by regressing their 3DMM coefficients respectively. Finally, they project the 3D facial landmarks of reconstructed face meshes onto the image level. Therefore, they obtain $q^{fuse}$, $q^{r}$, and $q^{low}$ where q denotes projected image-level facial landmarks.


$$\begin{array}{c} L_{shape}=\frac{\text{1}}{N}\sum_{n=\text{1}}^{N}{\left|\left|\overset{fuse}{\underset{n}{q}}-\overset{r}{\underset{n}{q}}\right|\right|}_{\text{1}}+{\left|\left|\overset{fuse}{\underset{n}{q}}-\overset{low}{\underset{n}{q}}\right|\right|}_{\text{1}} \end{array}$$ (11)

In our architecture, however, we do not consider low-resolution feature maps since they require overhead and can be complemented by our architecture where source information and target information are mutually blended across cross attention layers. Thus, we simplify shape loss as follows:


$$\begin{array}{c} L_{shape\;simplified}=\frac{\text{1}}{N}\sum_{n=\text{1}}^{N}{\left|\left|\overset{fuse}{\underset{n}{q}}-\overset{r}{\underset{n}{q}}\right|\right|}_{\text{1}} \end{array}$$ (12)

Where the supervision about shape using a 3D face model and a 2D landmark model performs to preserve a shape of a source face. With all the loss terms above, the overall loss can be written as:


$$\begin{array}{c} L=\lambda_{adv}*L_{adv}+\lambda_{attr}*L_{attr}+\lambda_{rec}*L_{rec}+\lambda_{id}*L_{id}+\;\lambda_{cont}*L_{cont}+\lambda_{lm}*L_{lm}+\lambda_{unet}*L_{unet}+\lambda_{splShape}*L_{splShape} \end{array}$$ (13)

## 4. Experimental results

### 4.1. Implementation details

We use Flickr Faces High Quality (FFHQ) [22], CelebFaces Attributes High-Quality (CelebA-HQ) [42], and Visual Geometry Group Face (VGGFace) [43] for training CrossSwap. The results in Tables 1 and 2 were measured as the output after training with this dataset. All comparison models used the same dataset. FFHQ is a high-quality dataset of 70,000 diverse human faces, with rich variations in age, ethnicity, and accessories like glasses and hats. CelebA-HQ is an enhanced version of the CelebA dataset with 30,000 celebrity images, offering high-resolution and quality that are beneficial for generating high fidelity face images. VGGFace is a large-scale dataset with over 3.3 million images of 9,000 + identities, designed for robust face recognition across different poses, lighting, and expressions. We trained our model with four NVIDIA RTX 3090 GPUs of 24GB Video Random Access Memory (VRAM), and it is verified the model is trainable using a single NVIDIA RTX 3090 GPU with a fewer batch size compared to our training settings.

**Table 1:** Performance on Pose, ID, FID, Expression metrics of CrossSwap and the baseline methods. ↓ indicates the lower, the better performance, and ↑indicates the higher, the better.

Method	Pose↓	ID↑	FID↓	EXP↓
CrossSwap	3.804	0.276	11.512	1.377
E4S	8.621	0.287	24.494	1.494
DiffFace	10.229	0.272	100.343	5.283
Ghost	6.824	0.270	8.758	1.706

**Table 2:** Comparison table of identity metric between ArcFace embedding and shape-aware identity embedding. The two models showed similar results in the identity preservation part.

Embedding Method	ID↑
ArcFace embedding	0.271
Shape-aware identity embedding	0.276

As the mentioned datasets are in different resolutions, we resize the images to 256 and then align and crop faces. To ensure high quality training, we manually sort out images that are low quality, with unlikely artifacts, and multiple faces in one image. Our test data is created with Faceforensics++ [44] which contains 1000 videos of different identities. We sample 10 frames from each video which results in 10,000 images in total.

In our experiment, we do not include landmark loss and shape loss for the first 2 epochs as landmark detection task is well done when face is reasonably generated. We set 7 features in scale from bottleneck until the final generated image feature and 6 AdaIN features in scale in our experiment where our model performs the best. As mentioned earlier, we do not calculate the last feature in the generator for the sake of GPU memory efficiency, which only activates transpose-convolution operation at the last feature scale.

### 4.2. Comparison with existing methods

Our method is compared with 4 different state-of-the-art methods including Encoder for Editing via StyleGAN (E4S) [27], DiffFace [4], Ghost [37], and DiffSwap [16]. With pre-made 10,000 test image frames from Faceforensics++ [44], we evaluate ID, FID, pose and, expression respectively. ID using ArcFace is a metric that measures the cosine similarity between feature embeddings extracted from images, evaluating how well a model can match the same person across images. This is useful in measuring how well the model preserves the identity of a source image after swapping faces, where a higher ID score indicates the model is more likely to be successful in creating realistic content. FID is defined by comparing the statistical distribution (mean and covariance) of real and generated image embeddings using a pretrained Inception network, assessing how realistic generated images are. In the FID measurement, the image is first transformed from a simple cluster of pixels into a high-dimensional feature distribution through the Feature Extraction process. Next, if the extracted feature vectors follow a Multivariate Gaussian Distribution, the mean and covariance of each distribution are calculated. In content creation, a lower FID score means that AI-generated images look closer to actual photos, making it valuable for producing engaging and authentic visuals in media and advertising. Pose refers to the orientation and configuration of a subject’s body, including the head, limbs, and torso, in 3D space. The generated limbs might be twisted in impossible ways, joints might be bent incorrectly, or the overall stance might look stiff, awkward, or physically implausible. The pose measurement considers a joint to be ‘accurate’ if the distance between the predicted joint position and the ground truth position is within a specific threshold. It represents the percentage of correctly generated joints out of the total joints. Expression refers to the dynamic changes in facial features (e.g., eyes, eyebrows, mouth, nose) that convey emotions or intentions. The expression is calculated by passing the generated image through a facial expression recognition model and using probability values (Softmax) for each emotion category (Happiness, Anger, Surprise, etc.). Overcoming pose and expression errors is crucial for generative AI to produce truly believable and controllable human-like content, which has wide-ranging implications for virtual reality, gaming, animation, and digital communication. We use the pre-trained weight of the other methods for inference.

Table 1 shows that our method is very competitive compared to all the other methods in most of the measured metrics. Although E4S [27] presents the best score in the ID metric, we reckon that the reason behind the superb result of E4S is because E4S uses their own re-coloring technique which fuses source face skin tone into target face attributes which leads swapped faces to acquire source face skin color. As to ID metric, we use the source face identity embedding and the swapped face embedding to calculate. Therefore, there is leaked information of source skin tone in source identity embedding which leads E4S to have the high score in Table 1. In summary, our method performs very competitive compared to the other methods in pose, ID, and expression metrics. Even though FID of CrossSwap is not the best among the methods, it still proves fair competitiveness in the metric. Ghost showed excellent results in ID and FID. However, it showed relatively low Pose and EXP scores. This means that Ghost’s output result has a similar face to the original image, but it is not natural and has frequent errors.

To qualitatively evaluate cross-attention, we additionally conducted comparative analysis experiments with three existing models. Experimental results show our method keeps the best with expression consistency and identity preservation. As the goal is to have the target face in the source face identity, the purpose is well served with ours while DiffFace [4] does not generalize well at all with our experiment. Ghost [37] generates unnecessary artifacts and does not keep mouth and teeth well. E4S [27] showcases generally good consistency. However, as it tries to fuse the source face color to the target face, the result is often unnatural. Compared to the other methods, our method keeps colors and shape without unnatural artifacts.

We experiment a few images of the same target and the source faces from the same identities to examine the consistency quality of the swapped results from the methods. The analysis reports that our method shows consistency in identity preservation along with the target face attributes. The source face has more prominent nasolabial folds and darker skin on the right upper cheek. These are well fused in the result of our method.

One of the most difficult challenges in face swapping is handling occlusions. Despite glasses as an occlusion in the target images, CrossSwap keeps them naturally by learning how to keep target attributes. Unlike our method, Ghost [37] almost neglects glasses which should be intact. Although Ghost still tries to generate glasses at its own best, it only adds unnatural artifacts around eyes. Unlike Ghost, E4S [27] keeps glasses in the target images in all their results. But it fails to keep consistency of hair in the example. Some results follow the hair of the target and some follow of the source. In addition, E4S suffers from inconsistency in colors below eyes. As mentioned, E4S tries to keep color of the source face which brings inconsistent results. Unfortunately, our method fails to be consistent in shape of lips on some occasions. The method clearly tries to keep the shape of the lips, but it still fails to be accurate occasionally although landmark loss aspects, which indicates that CrossSwap performs well in the wild with good consistency.

As we adopt an Identity extractor with 3D features and cross-attention mechanism for fusion, computational footprint is high. Due to this fact, we experiment various studies in the section of Ablation Study to see the trade-off of computational footprint and performance is reasonable. We see computational efficiency should be managed with focus. Rather than naively using pixels, utilizing patches with vision transformers would make the model more computationally efficient. Therefore, including approach and other computational optimization should be considered in future research.

Cross-Attention methods have shown powerful performance in deep learning, especially in transformer models, but they also have some drawbacks. The main drawbacks are as follows: Cross-attention takes two different sequences as input, so every element of one sequence interacts with every element of the other sequence and needs to compute the attention weights. Since the attention weight matrix needs to be stored, the memory requirement increases proportionally as the sequence length increases. This can be particularly problematic in environments with limited GPU memory, making it difficult to reduce the batch size or process longer sequences. Although the attention mechanism emphasizes important information through ‘focusing’, this also implies the possibility that ‘less important’ information may be ignored or lost. If the attention weights are focused on too few elements, the overall contextual information may be missed.

### 4.3. Ablation study

We show how the replacement of ArcFace that has been conventionally used as an identity embedding extractor with shape-aware identity embedding extractor leads to substantial improvement of identity metric. Recently many researchers found it may not be reasonable to use ArcFace as an identity embedding since ArcFace originally aimed to recognize human faces, not to extract identity information from them. We assume Shape-Aware Identity Embedding, which is a combination of ArcFace embedding and a mixed vector that consists of an identity coefficients source of and a pose and expression coefficients of target face, produces better swapped images in terms of human perception due to its richer information about the identity of source and inclusion of 3DMM coefficients of target. As a result of our ablation study between two different identity extractors.

Table 2 shows the use of shape-aware identity embedding brings significant improvement in ID evaluation score compared to ArcFace with the help of our cross-attention based architecture. Since the cross-attention generator attends to the global regions of each target and source face, abundant information of shape-aware identity embedding exerts its genuine power without a waste of information.

We test how the U-Net network could impact depending on training procedure differences. The reason behind this study comes with our early test results that show slow convergence during training. We assume one of the main reasons is the fact that the two U-Nets and the cross-attention generator are trained at the same time which burdens the whole training to slow down to converge. To accelerate the training procedure, we independently pre-train the U-net with partial datasets of VggFace2 [43]. The pre-trained U-Net reconstructs quickly as a standalone model which guarantee reconstruction ability in our test. This pre-trained U-Net is then used in CrossSwap without back-propagation weight update, which performs better and faster in CrossSwap. After two thousand iterations, the standalone pre-training U-net already reconstructs well the original input face whereas end-to-end U-net within CrossSwap is still not able to reconstruct the input properly. Indeed, the whole training of CrossSwap converges faster with an independently trained U-net model which makes the training procedure efficient in our experiments.

## 5. Conclusions

We propose CrossSwap which shows competitive results against various methods. CrossSwap can preserve the facial shape and rich identity features of source face while keeping attributes, for instance, pose of target face. We introduce cross-attention techniques to learn by leveraging cross-attention techniques, CrossSwap learns how to fuse the source, and the target faces which are empowered by our identity extractor in preservation of the source face identity. We conduct extensive quantitative and qualitative experiments with state-of-the-art methods. The experiments demonstrate CrossSwap is with excellence in many aspects in our evaluation metrics including ID, FID, Pose error, and Expression error.

CrossSwap also showcases the improvement in terms of shape awareness achieved by shape-aware identity extractor and shape loss and well-designed surrounding modules including cross attention-based generator, maintaining the best balance between the identity of source images and attributes of target images. Looking ahead, facilitating its application in creating photo-realistic swapped faces, CrossSwap has significant potential in industries such as film, gaming, and digital security. In the film industry, it could be used for realistic character face swapping, enhancing visual effects and enabling seamless scene transitions. In gaming, CrossSwap can allow for dynamic character customization, where faces can be swapped while maintaining natural identities and expressions. Future work may involve exploring how CrossSwap can be integrated with existing IT infrastructure, offering practical deployment strategies for these real-world applications.

The computational complexity of cross-attention is proportional to the square of the sequence length, which makes it computationally expensive for high-resolution images. To address this, instead of computing attention for all pixel pairs, we plan to reduce the computational complexity by focusing only on relevant pixel pairs using predefined patterns (e.g., computing only within a local window) or a learnable method. We also plan to reduce the computational complexity to be linearly proportional to the sequence length, which will improve the efficiency in large-scale image and video processing.

## Abbreviation:

3DMM: 3D Morphable model
CA: Cross-Attention
CelebA-HQ: CelebFaces Attributes High-Quality
E4S: Encoder for Editing via StyleGAN
EXP: Expression Errors
ID: Identity Retention
FFHQ: Flickr Faces High Quality
FFN: Feed-Forward Networks
FID: Fréchet Inception Distance
FSGAN: Face Swapping GAN
GAN: Generative Adversarial Network
GPU: Graphics Processing Unit
MAE: Masked AutoEncoder
MLP: Multilayer Perceptron
NLP: Natural Language Processing
Pose: Pose Errors
StyleGAN: Style-Based Generative Adversarial Network
VGG: Visual Geometry Group
VRAM: Video Random Access Memory
